# Effects of Total Dissolved Gas Supersaturation on the Swimming Performance of Two Endemic Fish Species in the Upper Yangtze River

**DOI:** 10.1038/s41598-018-28360-7

**Published:** 2018-07-03

**Authors:** Yuanming Wang, Yong Li, Ruidong An, Kefeng Li

**Affiliations:** State Key Laboratory of Hydraulics and Mountain River Engineering, Sichuan University, Chengdu, 610065 China

## Abstract

Total dissolved gas (TDG) supersaturation has been identified as one of the possible negative environmental effects of the construction of dams in the upper Yangtze River. Juvenile Chinese sucker and Prenant’s schizothoracin fish were selected to evaluate the impact of TDG supersaturation on the swimming performance of fish in the Upper Yangtze River. The critical swimming speeds (U_crit_) of Chinese sucker were 4.06, 2.83, 2.87, 2.68, and 2.29 BLs^−1^ at the TDG supersaturation levels of 100, 117, 122, 125 and 130%, respectively. The U_crit_ of Prenant’s schizothoracin were 7.38, 4.32, 3.98, and 3.74 BLs^−1^ at the TDG supersaturation levels of 100, 117, 125 and 130%, respectively. The burst swimming speed (U_burst_) of the two species also significantly declined with increases in the TDG supersaturation level. The present results demonstrate that the swimming speeds of Prenant’s schizothoracin that were exposed to 130% TDG supersaturation for 2 h exhibited significant recovery after 2 days, whereas the swimming speeds of Chinese sucker did not. The swimming speeds of Chinese sucker after 2 days of recovery were significantly reduced compared with those of control fish, whereas the speeds of Prenant’s schizothoracin returned to normal levels.

## Introduction

To meet the requirements of flood control and water supply, many dams have been built in the upper Yangtze River Basin^1–3^. The construction of dams results in the fragmentation of the upper Yangtze River and the destruction of the eco-hydrological conditions necessary for endemic fish survival. Total dissolved gas (TDG) supersaturation has been identified as one of the possible negative environmental effects of dam installations, and it threatens fish and other aquatic species^4,5^. During flood season, water is allowed to spill from the discharge structures of dams and entraps air in the plunge pools downstream from the dams, leading to increasing TDG in the downstream river^6–8^.

TDG supersaturation can easily cause fish to suffer from gas bubble disease (GBD) by producing emboli in the blood, heart and gill filaments. GBD signs in fish affected by TDG supersaturation have been observed in numerous rivers where dams have been constructed^9–12^. GBD can cause a variety of physiological impairments to fish and negatively impact their typical life processes^13^. Laboratory studies have demonstrated that fish swim more slowly with increases in their TDG supersaturation exposure time and may even lose their balance in supersaturated water^14,15^.

Swimming is an important physiological activity in fish that is closely related to their foraging behavior, evasion capacity and propagation^16^. Endemic fish in the upper Yangtze River need to pass dams with the help of fishways to migrate to spawning grounds, feeding grounds, or overwintering grounds. Good swimming performance is the key to successful migration. Fish swimming behavior can be classified into three major categories: sustained, prolonged, and burst swimming. Sustained swimming speed is the speed that can be maintained by fish for long periods, and that fueled aerobically. Prolonged swimming speed is of shorter duration than sustained, and ends in fatigue of the fish. Critical swimming (U_crit_) is a special category of prolonged swimming. Burst swimming speed (U_burst_) is the highest speed of which fishes are capable, and can be maintained only for short periods^17,18^.

With the construction of high-head dams on the upper Yangtze River Basin, the swimming performance of endemic fish downstream from the dams may be affected by TDG supersaturation. Resident fish stressed by TDG supersaturation may be considerably more vulnerable to predators, and their foraging behavior may also worsen due to their decreased swimming capabilities. In addition, it may also become more difficult for fish to pass through or over dams when they are stressed by TDG supersaturation. However, previous studies have mainly focused on the lethal effects of TDG supersaturation on fish^14,19–22^, and some researchers have assessed the detection and avoidance characteristics of fish in supersaturated water^14,23^. At present, the impact of TDG supersaturation on the swimming performance of fish in the upper Yangtze River has attracted little attention.

Chinese sucker *Myxocyprinus asiaticus* and Prenant’s schizothoracin *Schizothorax prenanti* are both endemic species in the upper Yangtze River^24^. Chinese sucker, the only species of Catostomidae found in China^25^, was recently listed in the China Red Data Book of Endangered Animals^26^. The population of Prenant’s schizothoracin has also greatly declined in recent years, and this decline is associated with dam construction^27^.

To study the impact of TDG supersaturation on the swimming performance of fish in the upper Yangtze River, the U_crit_ and U_burst_ values of Chinese sucker and Prenant’s schizothoracin, the experimental fish, were assessed in individuals exposed to TDG supersaturated water. The results of this study can be used to inform the operation of hydropower stations and contribute fundamental data for the establishment of water-related environmental standards in China.

## Materials and Methods

### Ethical statement

The animal study proposal was approved by the Ethics Committee for Animal Experiments of Sichuan University. All experimental procedures were performed in accordance with the Regulations for the Administration of Affairs Concerning Experimental Animals approved by the State Council of the People’s Republic of China.

### Experimental fish

Healthy juvenile Chinese sucker and Prenant’s schizothoracin fish obtained from the Sichuan Fisheries Research Institute of China were used in this study. The fish were maintained in an air-equilibrium (100% TDG) water pool for 7 days before the experiment. The water temperature ranged from 23–25 °C. The dissolved oxygen (DO) level was maintained from 7.0–7.6 mg/L, and the pH range was 7.5–7.8. During the acclimation period, fish were fed Tubificidae twice daily, and the feeding was stopped 24 hours before the experiment to avoid impacts caused by the fish food.

### Generation of TDG supersaturated water

**Figure 1:**
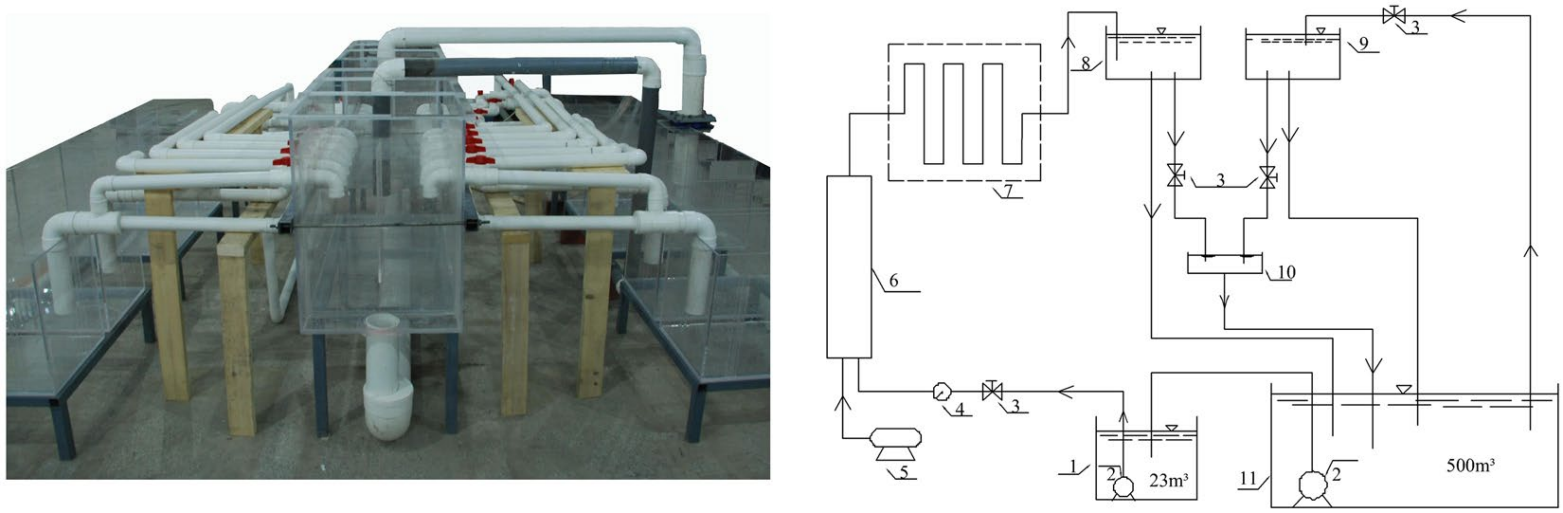
Photograph and schematic diagram of the circulating system for the TDG supersaturated water generation. 1: water pool, 2: pumps, 3: valves, 4: meter, 5: compressor, 6: pressure vessel, 7: steel pipe, 8: TDG supersaturation tank, 9: saturated-equilibrium water tank, 10: experimental tank, and 11: cistern.

A circulating system, which included a large cistern (11) with a total air-equilibrium water volume of 500 m^3^, was used to generate the TDG supersaturated water (Fig. 1). Air equilibrium water was first imported to the water pool (1) and then introduced to a vessel with compressed air (6). Under the high pressure from the pump (2), air dissolved in the water within the steel pipe (7), which resulted in TDG supersaturation. The supersaturated water was then introduced to the tank (8). By mixing a certain amount of air equilibrium water with the supersaturated water in the experimental tank, the TDG level could be controlled. In addition, the gas level was monitored by a point-four tracker (Point Four Systems Inc., Canada) at the surface of the tank (10).

### Experimental procedure

After 7 days of acclimation, a total of 70 Chinese sucker and 65 Prenant’s schizothoracin fish were introduced into 35-cm deep tanks with the TDG supersaturated water for 2 h of exposure. The selected levels of TDG supersaturation ranged from 100 to 130%, which is a range commonly found downstream from dams on the upper Yangtze River Basin^7,28^. The exposure length of 2 h to TDG supersaturation less than 130% has been demonstrated to be sublethal for most fish^14,22,29^. Five TDG supersaturation levels (100, 117, 122, 125 and 130% TDG) were used for the Chinese sucker, and four TDG supersaturation levels (100, 117, 125 and 130% TDG) were used for the Prenant’s schizothoracin. At each gas level, 4 to 6 fish were first exposed to the TDG supersaturated water for 2 h and then immediately transferred to a swim tunnel respirometer to test their U_crit_ or U_burst_.

In addition, 22 Chinese sucker and 25 Prenant’s schizothoracin fish were placed into 130% TDG supersaturated water for 2 h and then transferred to the air equilibrium water pool for 2 days for recovery. Then, the swimming performance of the fish was assessed by testing the U_crit_ and U_burst_ values. The experimental fish were removed immediately if they died during the TDG supersaturation exposure process or the recovery process.

As a control, the U_crit_ and U_burst_ of healthy Chinese sucker and Prenant’s schizothoracin fish (0 h of TDG supersaturation exposure) were also measured. The body mass and body length of the fish used in the U_crit_ and U_burst_ test are presented in Tables 1 and 2. For the tests, each fish was used only once.

**Table 1:** The body mass and body length of juvenile Chinese sucker used in the U_crit_ and U_burst_ test (the data are expressed as the mean ± S.E., *n* = 4–6).

Chinese sucker		Treatment conditions					
		100%	118%	122%	125%	130%	Recovery
U_crit_	n	6	4	4	4	6	4
	Body mass (g)	32.2 ± 2.8	30.5 ± 0.7	28.8 ± 1.7	30.3 ± 3.0	32.3 ± 2.5	29.3 ± 1.4
	Body length (cm)	13.4 ± 0.4	13.4 ± 0.5	13.1 ± 0.4	13.5 ± 0.5	13.4 ± 0.4	13.1 ± 0.4
	Speed (BL/s)	4.06 ± 0.18	2.83 ± 0.22	2.87 ± 0.31	2.68 ± 0.36	2.29 ± 0.25	3.07 ± 0.17
U_burst_	n	6	4	4	4	6	4
	Body mass (g)	31.0 ± 2.5	30.8 ± 0.8	27.3 ± 0.5	27.3 ± 1.8	28.3 ± 0.8	27.0 ± 0.4
	Body length (cm)	14.2 ± 0.5	13.7 ± 0.2	13.0 ± 0.5	13.0 ± 0.2	12.7 ± 0.4	12.9 ± 0.1
	Speed (BL/s)	4.34 ± 0.24	3.25 ± 0.16	3.26 ± 0.29	3.32 ± 0.46	2.87 ± 0.20	3.42 ± 0.17

The column title “Recovery” refers to the condition where the fish were exposed to 130% TDG for 2 h and then allowed 2 days of recovery. Each fish was used only once. Body length was analyzed with one-way ANOVA, and no significant difference in body length was found in the U_crit_ and U_burst_ groups.

**Table 2:** The body mass and body length of the juvenile Prenant’s schizothoracin used in the U_crit_ and U_burst_ test (the data are expressed as the mean ± S.E., *n* = 4–6).

Prenant’s schizothoracin		Treatment conditions				
		100%	118%	125%	130%	Recovery
U_crit_	n	6	5	5	6	6
	Body mass (g)	9.5 ± 1.3	10.2 ± 0.6	10.6 ± 0.8	10.0 ± 0.6	9.8 ± 0.6
	Body length (cm)	9.9 ± 0.4	10.9 ± 0.4	11.3 ± 0.6	11.3 ± 0.2	10.3 ± 0.4
	Speed (BL/s)	7.38 ± 0.44	4.23 ± 0.16	3.98 ± 0.45	3.74 ± 0.11	6.75 ± 0.32
U_burst_	n	5	4	4	5	6
	Body mass (g)	10.2 ± 0.6	11.0 ± 0.7	11.0 ± 0.4	10.2 ± 0.4	10.0 ± 0.5
	Body length (cm)	10.5 ± 0.3^b^	10.9 ± 0.1^ab^	11.8 ± 0.5^b^	10.1 ± 0.2^b^	10.4 ± 0.4^b^
	Speed (BL/s)	7.52 ± 0.14	4.82 ± 0.19	4.46 ± 0.25	4.66 ± 0.23	7.18 ± 0.33

The column title “Recovery” refers to the condition where the fish were exposed to 130% TDG for 2 h and then allowed 2 days of recovery. Body length was analyzed using one-way ANOVA, and data that do not share a common letter are significantly different (*P* <0.05). No significantly differences in body length were found in the U_crit_ group.

### Measurement of U_crit_ and U_burst_

**Figure 2:**
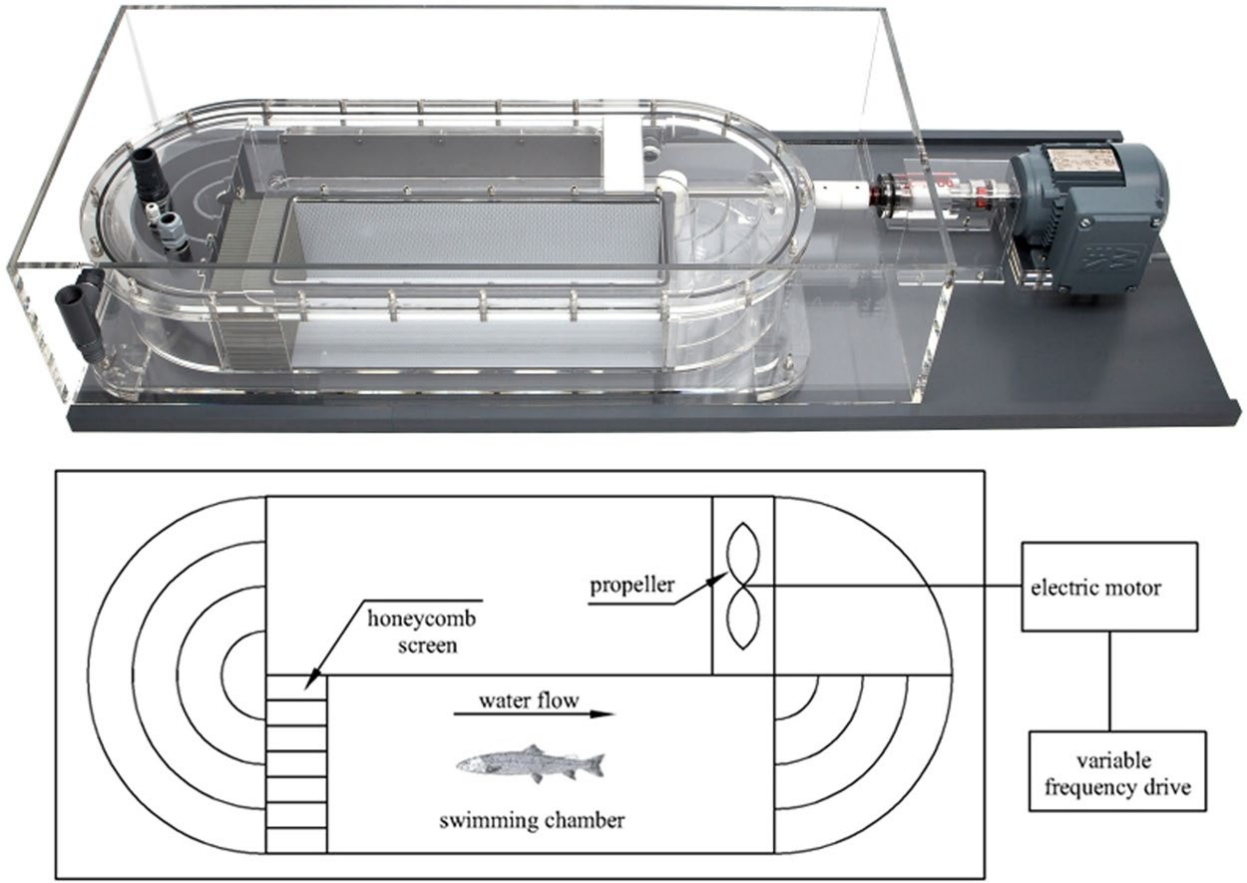
Photograph and schematic diagram of the swim tunnel respirometer (Loligo Systems SW10200, Denmark) used to test the swimming performance of the Chinese sucker and Prenant’s schizothoracin. The authors drew the fish.

The U_crit_ and U_burst_ were measured in a swim tunnel respirometer (Loligo Systems SW10200, Denmark) with a 90 L water volume (Fig. 2). A circulating water flow was generated in the tunnel with a single propeller powered by an electric motor with variable frequency drive. The swimming chamber of the tunnel was 70 × 20 × 20 cm (length × width × height), and a honeycomb screen was installed at the upstream area to reduce the turbulence and ensure that the water had uniform velocity as it passed through the swimming chamber. The water velocity in the swimming chamber was controlled within a wide range (5–150 cm s^−1^), and measured by a propeller flow meter. The water temperature in the swimming chamber ranged from 23 to 25 °C, and the DO concentration ranged from 7.0 to 7.6 mg/L.

The U_crit_ and U_burst_ were determined by using the velocity increasing method^30^. Fish under the TDG supersaturation exposure were first placed into the swimming chamber with 5 cm s^−1^ flow for 20 min to eliminate the stress effect of the transfer process, and the U_crit_ test began with an increase in the water velocity from the 5 cm s^−1^ acclimation velocity to 50% of the estimated U_crit_^31,32^. The water velocity in the swimming chamber was then steadily increased in 10 cm s^−1^ increments every 20 min until the fish were exhausted. Normally, the duration of the test process was limited to 2 h. The U_crit_ was calculated as follows:

1 $${U}_{crit}=V+(t/T){\rm{\Delta }}V$$

where $V$ is the highest velocity maintained for the entire time interval (cm s^−1^); ${\rm{\Delta }}V$ is the velocity increment (10 cm s^−1^); $T$ is the time interval at each swimming speed (20 min); and $t$ is the time that the fish swam at the exhaustion velocity (min). The solid blocking effect was considered to be negligible because the volume of the fish was <10% of the swim chamber^33^. All absolute U_crit_ (cm s^−1^) values were converted to body length per second, which denotes the relative U_crit_ (BL s^−1^).

The U_burst_ test was similar to the U_crit_ test. Fish were held in the swimming chamber for 20 min at a water speed of 5 cm s^−1^ during the acclimation period. Then, the U_burst_ test began with an increase in the acclimation water velocity to 50% of the estimated U_burst_. The water velocity in the swimming chamber was then steadily increased in 10 cm s^−1^ increments every 1 min until the fish were exhausted.

### Statistical analysis

A two-way analysis of variance (ANOVA) was performed to determine the effects of the fish species, the TDG supersaturation level and their interaction on swimming speeds (both U_crit_ and U_burst_). One-way ANOVA was used to test whether the swimming speeds at different TDG supersaturation levels were significantly different. Body length was also analyzed with one-way ANOVA considering it has a profound effect on swimming performance. Post hoc multiple comparisons were performed using the Least Significant Difference tests whenever the data displayed homogeneous variance, whereas Dunnett’s T3 test was used when non-homogeneous variance was noted (by Levene’s test). Regression analysis was used to estimate the relationship between the swimming speeds and TDG supersaturation levels. The date difference between two experimental groups was analyzed by independent samples t-test. The level of significance for the differences was *P* <0.05.

## Results

### Effect of TDG supersaturation on fish

After 0.5 h of exposure, the fish at the 130% TDG supersaturation level were maladjusted to the TDG supersaturated water (some fish lost their balance and fell to the bottom of the experiment tanks, and some jumped up and hit the nets in the upper part of the tanks), whereas fish at the other TDG supersaturation levels seemed to exhibit no abnormal behaviors. All the fish had evident gas bubbles on their fins, but no fish died after the 2-h exposure. When the fish exposed to 130% TDG supersaturation for 2 h were transferred to the air equilibrium water pool, they either swam slowly or remained stationary at the bottom of the pool. Some of the fish moved up to the surface and released gas bubbles from their mouths. Two out of 22 Chinese sucker and 4 out of 25 Prenant’s schizothoracin died within 12 h in the recovery pool. In addition, the gas bubbles on the remaining fish disappeared gradually, and the fish exhibited normal behavior after 2 days of recovery.

### Swimming performance of fish under TDG supersaturation stress

Both the fish species and TDG supersaturation level significantly affected the U_crit_ and the U_burst_ (Table 3). The largest contributor to the total variation of U_crit_ was TDG, while the fish species and their interaction followed. Regarding the total variation of U_burst_, fish species was the largest contributor, while TDG and their interaction followed.

**Table 3:** Statistical analysis of two-way ANOVA on the U_crit_ and the U_burst_ of fish.

Sources of Variation		df	Mean square	F	sig.	Partial eta squared
U_crit_	TDG	4	13.11	28.48	<0.001	0.755
	Fish species	1	33.37	72.44	<0.001	0.662
	Fish species * TDG	3	2.89	6.27	0.002	0.337
U_burst_	TDG	4	8.31	29.06	<0.001	0.779
	Fish species	1	33.96	118.83	<0.001	0.783
	Fish species * TDG	3	1.94	6.77	0.001	0.381

**Figure 3:**
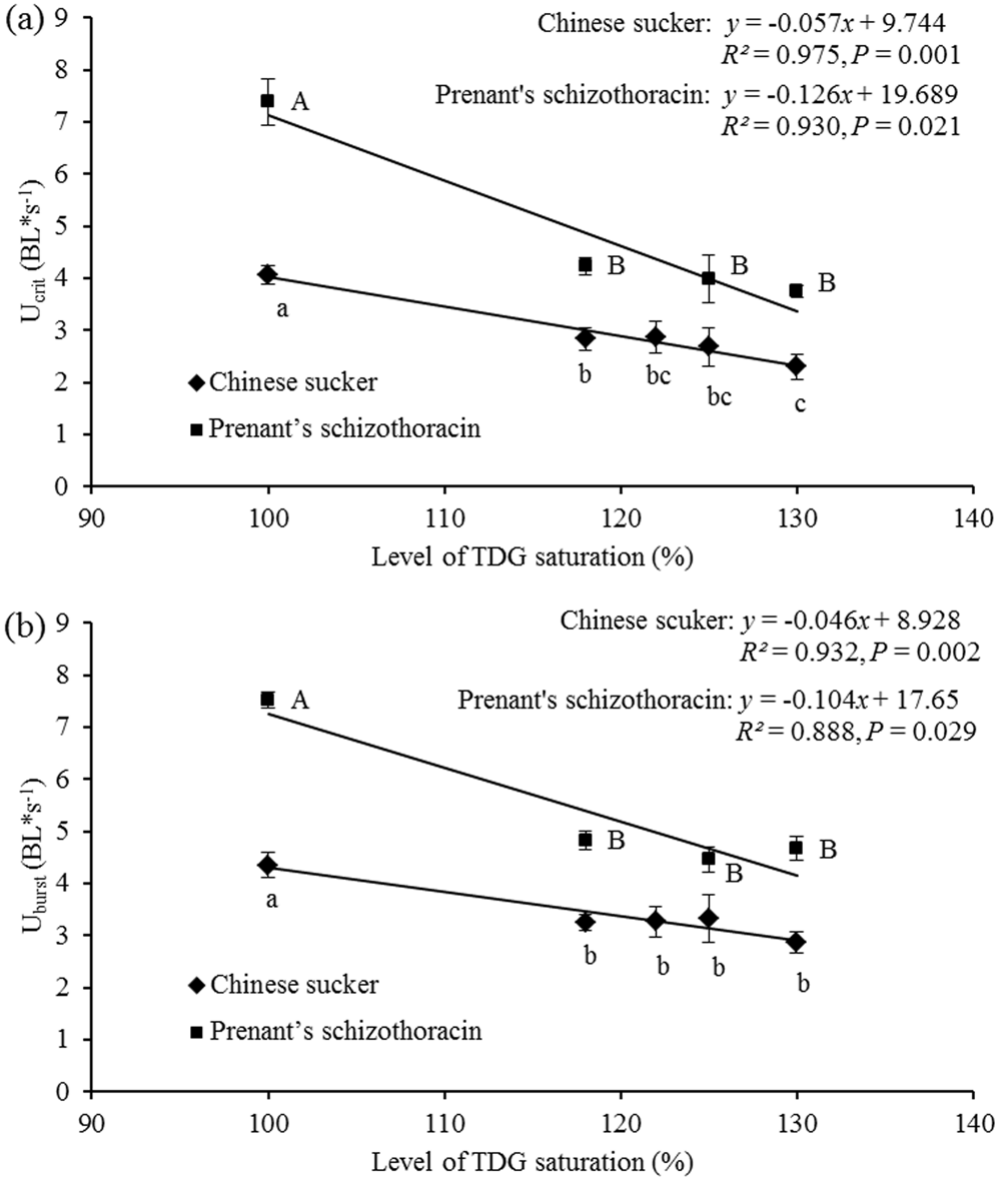
The swimming speeds of the experimental fish vary with the TDG supersaturation level. (**a**) U_crit_ vs. TDG supersaturation level. (**b**) U_burst_ vs. TDG supersaturation level. Values are presented as the mean ±S.E. (*n* = 4–6). Letters following the means indicate the results of a post hoc multiple comparison test (least significant difference test); means that do not share a common letter are significantly different (*P* <0.05).

The U_crit_ of the fish significantly decreased with TDG supersaturation (one-way ANOVA for Chinese sucker: F_(4,19)_ = 7.99, *P* = 0.01; Prenant’s schizothoracin: F_(3,18)_ = 28.55, *P* <0.001), decreasing by greater than 30% after the exposure to TDG supersaturated water. The U_crit_ values had a negative linear relationship with the TDG supersaturation level. The linear equation for Chinese sucker was *y* = −0.057 *x* + 9.744 (*R*^2^ = 0.975, *P* = 0.001) for Chinese sucker, and *y* = −0.126 *x* + 19.690 (*R*^2^ = 0.930, *P* = 0.021) for Prenant’s schizothoracin, where *y* is the U_crit_ value, *x* is the level of TDG supersaturation, and *R*^2^ is the regression coefficient (Fig. 3a). The U_crit_ values of Chinese sucker were significantly reduced compared with those of Prenant’s schizothoracin at each TDG supersaturation level, except for 125% (independent t-test: *P* = 0.07). However, the U_crit_ of Chinese sucker was 67% the value of the Prenant’s schizothoracin U_crit_ at the 125% saturation level.

The U_burst_ of the two types of fish significantly declined with decreasing TDG supersaturation (one-way ANOVA for Chinese sucker: F_(4,19)_ = 5.03, *P* = 0.006; and for Prenant’s schizothoracin: F_(3,14)_ = 53.05, *P* <0.001). A negative linear relationship was noted between the U_burst_ and the TDG supersaturation level (Fig. 3b). The U_burst_ values of Chinese sucker were significantly reduced at each TDG supersaturation level compared with those of Prenant’s schizothoracin, except for the 125% level (independent t-test: *P* = 0.07).

### Swimming performance of fish after recovery

**Figure 4:**
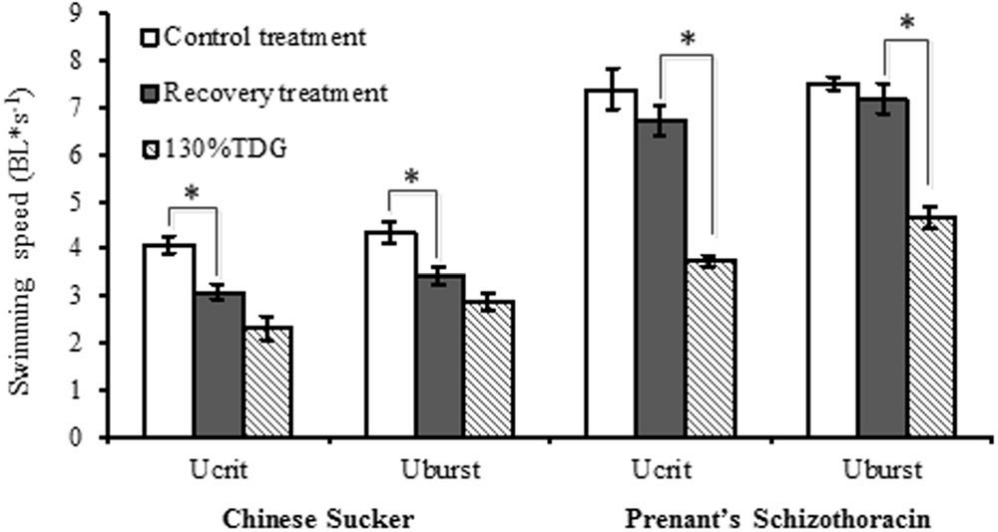
Swimming speed of experimental fish exposed to 130% TDG for 2 h and then allowed to recover for 2 d before testing. The swimming speeds of the control fish and the fish exposed 130% TDG for 2 h are presented for comparison. Values are presented as the mean ± S.E. (*n* = 4–6). *Significant difference in swimming speed between treatments (*P* <0.05).

The swimming speeds (both the U_crit_ and U_burst_) of Chinese sucker were less than 50% of those of Prenant’s schizothoracin. The swimming ability of the fish that were exposed to the 130% TDG supersaturation condition for 2 h improved after the 2-day recovery period (Fig. 4). The U_crit_ and U_burst_ of Chinese sucker increased by 34 and 19% after 2 days of recovery; however, the increases were not significant (independent T-test analysis for U_crit_: F_8_ = 0.741, *P* = 0.052; and for U_burst_: F_8_ = 1.129, *P* = 0.08). The swimming speeds of Prenant’s schizothoracin significantly increased after 2 days of recovery (*P* <0.05) by greater than 50%. After recovering for 2 days, the U_crit_ and U_burst_ of Chinese sucker that experienced 2 h of 130% TDG exposure reached 76 and 79%, respectively, compared with those of control Chinese sucker, and the differences were significant (independent T-test analysis for U_crit_: F_8_ = 1.693, *P* = 0.005; U_burst_: F_8_ = 0.62, *P* = 0.024). The U_crit_ and U_burst_ of Prenant’s schizothoracin after 2 days of recovery reached 91 and 95% of the control U_crit_ and U_burst_, respectively. The differences in the U_crit_ and U_burst_ values of the recovery treatment and the control treatment were not significant (independent T-test analysis for U_crit_: F_10_ = 0.09, *P* = 0.27; U_burst_: F_10_ = 3.451, *P* = 0.396).

## Discussion

### Effect of TDG supersaturation on fish

Many environmental factors could affect the swimming performance of fish, such as water temperature, the fish feeding characteristics or water flow pattern^34–38^. Previous researchers have demonstrated that the dissolved gases in water can influence the swimming performance of fish. However, previous studies have mainly focused on a single gas (O_2_ or CO_2_). At present, TDG supersaturation has received minimal attention^34,35,38^. TDG supersaturation can cause emboli in gill blood vessels, which block the flow of blood, disrupting the function of organs and impairing the neural activity of fish^13^. In addition, gas bubbles in supersaturated water could also adhere to the skin of fish and make them lose balance and swim slowly^14,15^.

No fish died during the TDG supersaturation process in the present study, but all fish had evident gas bubbles; this finding is consistent with the results of previous studies^14,22^. Every year, Chinese sucker and Prenant’s schizothoracin in the upper Yangtze River need to migrate upstream to spawn by using their critical swimming speed, and they also need to use burst swimming to forage or escape from threats. With the construction of high dams in the upper Yangtze River, the swimming performances of Chinese sucker and Prenant’s schizothoracin are potentially affected by TDG supersaturation.

### Swimming performance of fish under TDG supersaturation stress

After 2 h of TDG supersaturation exposure, the critical and burst swimming speeds of the fish both considerably decreased. Previously, only a few studies have focused on the impact of TDG supersaturation on the swimming performance of fish. Schiewe (1974) reported that swimming capability of juvenile chinook salmon *Oncorhynchus tshawytscha* (mean weight 16.0 g, mean fork length 11.8 cm) decreased when they were exposed to 117 and 120% TDG supersaturation levels for greater than 10 h^39^. A significant decrease was only observed at the 120% TDG supersaturation level, and the chinook salmon suffered 10% mortality during the exposure process. Dawley & Ebel (1975) exposed steelhead trout *Oncorhynchus mykiss* to 120 and 125% supersaturated water until 10 or 50% mortality was reached. Then, the swimming performance of the survivors was assessed^40^. Their swimming performance was not significantly different compared with that of the control fish. The swimming speeds of the Prenant’s schizothoracin and Chinese sucker in this study were more easily affected by TDG supersaturation compared with those of chinook salmon and steelhead trout. However, it should be noted that the swimming performances of the chinook salmon and steelhead trout were determined by measuring the distance gained and swimming time against a constant water current, and the U_crit_ and U_burst_ of the fish were not strictly tested.

Schiewe (1974) also examined the swimming capability of juvenile chinook salmon exposed to 104, 106 and 112% TDG supersaturated water for 35 days, and no significant difference was noted between the control group and treatment group^39^. Similarly, the swimming capability of juvenile steelhead trout exposed to 105, 110 or 115% TDG for 35 days did not significantly differ from that of the control fish^39^. The swimming performance of Prenant’s schizothoracin and Chinese sucker exposed to water with lower (100–115%) TDG supersaturation levels should be further studied.

**Figure 5:**
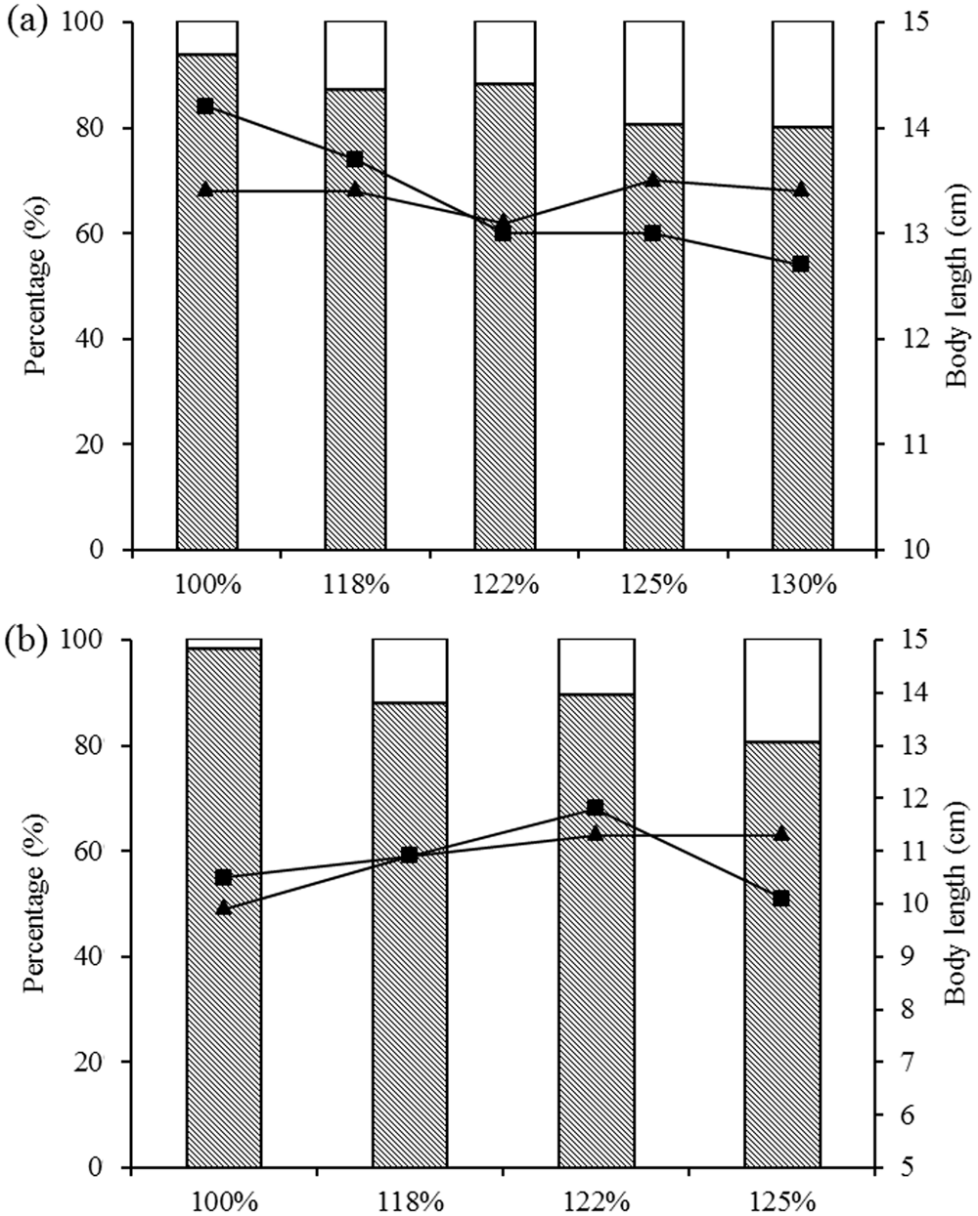
The proportion of experimental fish’s critical and burst swimming speed at different TDG levels. The U_crit_ data are presented as a percent of the U_burst_ data. Polyline with triangle represents body length of fish in the U_crit_ group, and polyline with square represents body length of fish in the U_burst_ group. (**a**) presents data from Chinese sucker, and (**b**) presents data from Prenant’s schizothoracin.

The present results demonstrated that the U_crit_ values of Chinese sucker and Prenant’s Schizothoracin were similar with the U_burst_ values. As shown in Fig. 5, the U_crit_ values of Chinese sucker reached 80–94% of the U_burst_ values at different TDG supersaturation level, and the U_crit_ values of Prenant’s schizothoracin were 80–98% of the U_burst_ values. In particular, the U_crit_ values of Chinese sucker and Prenant’s Schizothoracin at the 100% TDG level reached 94 and 98% of the U_burst_ values. This result was inconsistent with the findings of previous reports. The U_crit_ of Largemouth bass *Micropterus salmoides*, Atlantic cod *Gadus morhua* and Rainbow trout *Onchorhynchus mykiss* only reached 56–83% of their U_burst_^40–42^. We hypothesize that the body length of fish may mainly cause such a difference. The body lengths of fish in the U_crit_ group were larger compared with those of the U_burst_ group at the 100% TDG level.

### Swimming performance of fish after recovery

The present results indicate that most Chinese sucker and Prenant’s schizothoracin recovered from TDG supersaturation stress within 2 days in air equilibrium water, which is consistent with the results of Dawley & Ebel (1975) and Wang *et al*. (2015)^15,43^. After the chinook salmon and steelhead trout were exposed to 120, 125, and 130% TDG supersaturated water until 50% mortality was reached, they were allowed to recover at 100% gas saturation for up to 15 days. All GBD signs disappeared during the recovery process^43^. Wang *et al*.^15^ placed rock carp in 130% supersaturated water for 2.33 h and then held them in air equilibrium (100% TDG) water for 5 days^15^. They reported that these fish exhibited a poorer tolerance to TDG supersaturated water compared with that of control fish, whereas their horizontal and vertical behaviors exhibited no significant differences.

The swimming performance of fish always recovers from environmental stress after a considerable time^31,44,45^. This study demonstrated the swimming speeds of Prenant’s schizothoracin that were exposed to 130% TDG supersaturation for 2 h exhibited significant recovery after 2 days, whereas Chinese sucker did not. The swimming speeds of Chinese sucker after 2 days of recovery were significantly reduced compared with those of control fish, whereas the speeds of Prenant’s schizothoracin returned to normal levels. This finding indicates that the swimming performance of Prenant’s schizothoracin recovers from TDG supersaturation more easily compared with that of Chinese sucker. Such a difference may be caused by their habits and lifestyles. Chinese sucker always swim gently and require abundant oxygen. Conversely, Prenant’s schizothoracin is a cold-water fish and prefer turbulent water. Prenant’s schizothoracin are more active compared with Chinese sucker^46^.

The swimming distance and swimming time of the juvenile chinook salmon against a constant water current could be completely recovered when the surviving fish were held for 2 h in air equilibrium (100% TDG) water, after the fish were exposed to 120% TDG supersaturated water until a 50% mortality level was reached^38^. Therefore, it seems that the swimming capabilities of the chinook salmon examined by Schiewe (1974) recovered after exposure to TDG supersaturation conditions more easily compared with two types of fish examined in this study^39^.

## Conclusion and Suggestion

The TDG supersaturation problem of the upper Yangtze River Basin is becoming increasingly serious with the construction of high dams, and the fish downstream from the dams are threatened^7,8^. The results of this study provide important information that can help develop methods to protect fish threatened by TDG supersaturation conditions and guide operational processes at hydropower stations. The U_crit_ and U_burst_ of two endemic fish species in the upper Yangtze River Basin were significantly reduced as TDG supersaturation increased. The swimming speeds of Prenant’s schizothoracin exposed to 130% TDG supersaturation for 2 h exhibited a significant recovery after 2 days, whereas the swimming speeds of Chinese sucker did not. The swimming speeds of Chinese sucker after 2 days of recovery were significantly reduced compared with those of control fish, whereas the speeds of Prenant’s schizothoracin returned to normal levels.

Measures should be taken to protect the fish downstream from dams. Discharging floods discontinuously has proven to be an effective strategy to diminish the TDG supersaturation downstream from dams, allowing fish to have sufficient time to recover^47^. Politano *et al*.^48^ suggested that concentrating the spillway flow in one bay (especially in a central spillway bay) caused bubbles to travel closer to the free surface and thus reduced TDG production and enabled more degasification^48^. Additionally, the design and construction of fish passage facilities should consider the impact of TDG supersaturation on fish in the future^49^.
